# In Vitro and In Vivo Antimalarial Activity of LZ1, a Peptide Derived from Snake Cathelicidin

**DOI:** 10.3390/toxins11070379

**Published:** 2019-06-30

**Authors:** Yaqun Fang, Xiaoqin He, Pengcheng Zhang, Chuanbin Shen, James Mwangi, Cheng Xu, Guoxiang Mo, Ren Lai, Zhiye Zhang

**Affiliations:** 1 College of Life Sciences, Nanjing Agricultural University, Nanjing 210095, China; 2 Key Laboratory of National Health and Family Planning Commission on Parasitic Disease Control and Prevention, Jiangsu Provincial Key Laboratory on Parasite and Vector Control Technology, Jiangsu Institute of Parasitic Diseases, Wuxi 214064, China; 3 Key Laboratory of Bioactive Peptides of Yunnan Province/Key Laboratory of Animal Models and Human Disease Mechanisms of Chinese Academy of Sciences, Kunming Institute of Zoology, Kunming 650223, China

**Keywords:** antimicrobial peptide, malaria, cytokine, pyruvate kinase, ATP

## Abstract

Antimalarial drug resistance is an enormous global threat. Recently, antimicrobial peptides (AMPs) are emerging as a new source of antimalarials. In this study, an AMP LZ1 derived from snake cathelicidin was identified with antimalarial activity. In the in vitro antiplasmodial assay, LZ1 showed strong suppression of blood stage *Plasmodium falciparum* (*P. falciparum*) with an IC50 value of 3.045 μM. In the in vivo antiplasmodial assay, LZ1 exerted a significant antimalarial activity against *Plasmodium berghei* (*P. berghei*) in a dose- and a time- dependent manner. In addition, LZ1 exhibited anti-inflammatory effects and attenuated liver-function impairment during *P. berghei* infection. Furthermore, by employing inhibitors against glycolysis and oxidative phosphorylation in erythrocytes, LZ1 specifically inhibited adenosine triphosphate (ATP) production in parasite-infected erythrocyte by selectively inhibiting the pyruvate kinase activity. In conclusion, the present study demonstrates that LZ1 is a potential candidate for novel antimalarials development.

## 1. Introduction

Human malaria is caused by parasites of the genus plasmodium, of which *Plasmodium falciparum* (*P. falciparum*) is the most virulent and prevalent malaria parasite in nearly 100 malaria-endemic countries. According to the World Health Organization (WHO) [1], an estimated 219 million cases of malaria occurred worldwide in 2017. Although artemisinin-based combination therapy (ACT) has been recommended as first-line treatment for uncomplicated *P. falciparum* malaria since 2002 [2], *P. falciparum* resistance to artemisinin is currently emerging in the Greater Mekong Subregion [3]. Since antimalarial drug resistance poses a major threat to malaria control with important implications to global public health, development of new drugs to replace those that have become ineffective is paramount.

Natural resources from both invertebrates and vertebrates represent a rich source of bioactive compounds [4,5]. Antimicrobial peptides (AMPs) are usually defined as the first defense molecules produced by organisms in response to various infections [6], and they are being widely developed and show the potential advantage of being much less prone to resistance than the drugs currently in use [7,8]. Several AMPs-derived antimalarial peptides, such as cecropins [9], gambicin [10], and scorpine [11], have been proven to affect the life cycle of the malarial parasite at different stages. The human cathelicidin LL-37 has also been reported to possess antimalarial activity against the asexual blood stage of the *Plasmodium yoelii* parasite [12]. A cathelicidin named cathelicidin-BF was isolated from the venom of *Bungarus fasciatus* in our previous work [13], and it showed strong antibacterial activities against Gram-negative and Gram-positive bacteria [13,14]. Based on cathelicidin-BF, a panel of AMPs with enhanced antimicrobial activities was designed [15,16]. The designing strategies include substitutions of charged or hydrophobic amino acid residues for noncharged polar residues to promote antimicrobial activity and insertion of a hydrophobic residue in the hydrophilic side of the helix structure to reduce hemolysis. In this study, we found one of the designed AMPs named LZ1 exerted anti-plasmodium activity in vitro and in vivo by modulating host immune response and destroying intraerythrocytic parasite glucose metabolism.

## 2. Results

### 2.1. In Vitro Anti-Plasmodial Activity of LZ1

**Figure 1:**
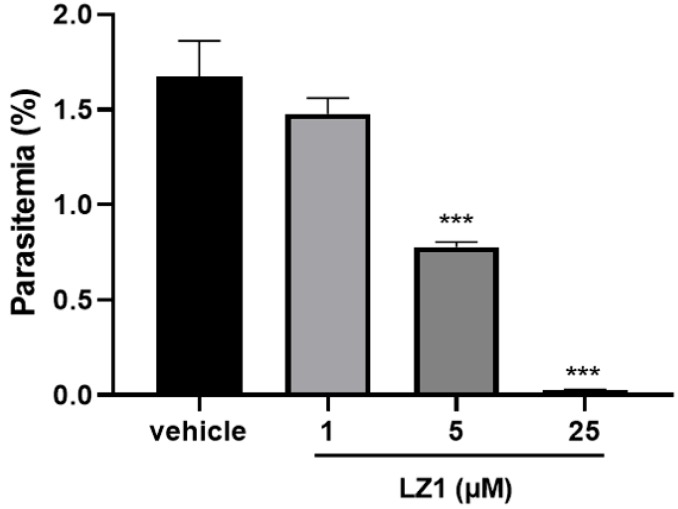
In vitro antimalarial activity of LZ1. In vitro antimalarial activity of LZ1 against the asexual blood stage of *Plasmodium falciparum* (*P. falciparum*). Data are presented as mean ± SEM. *** *p* < 0.001.

In our previous study, LZ1 exhibited promising antimicrobial activities against pathogens that are associated with acne vulgaris [15]. Here, we found that LZ1 exerts anti-plasmodial activity. To determine its antimalarial activity, in vitro antimalarial assay against *P. falciparum* line 3D7 was first evaluated. As illustrated in Figure 1, we examined its activity against the asexual blood stage of *P. falciparum* parasite. LZ1 showed significant suppression of parasitemia in a dose-dependent manner with a suppression ratio of 98.8% at a concentration of 25 μM. The IC50 value of LZ1 against *P. falciparum* was 3.045 μM. In addition, LZ1 caused a negligible hemolytic activity to human red blood cells (RBCs) [15].

### 2.2. In Vivo Anti-Plasmodial Activity of LZ1

**Figure 2:**
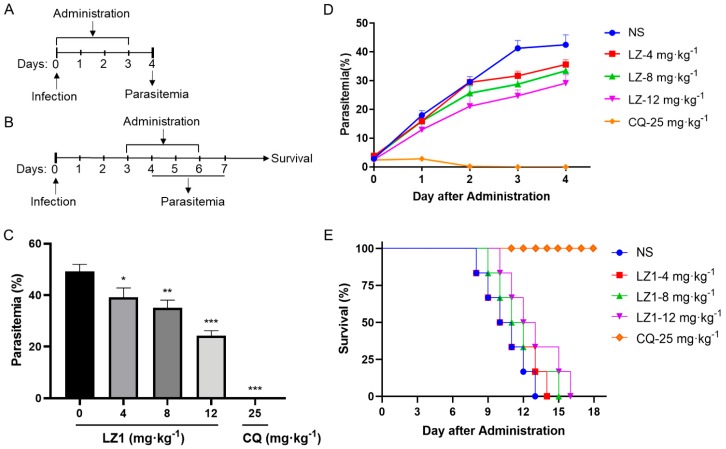
In vivo antimalarial activity of LZ1 against *Plasmodium berghei* (*P. berghei*). (**A**) Schematic of the four day suppression test. (**B**) Schematic of Rain’s test. (**C**) Parasitemia of different group was shown in the four day suppression test. (**D**) Parasitemia of a different group was shown in Rain’s test. (**E**) Survival rates were determined in Rain’s test. NS, normal saline. CQ, chloroquine. Data are presented as mean ± SEM (*n* = 6 mice per group). * *p* < 0.05, ** *p* < 0.01, *** *p* < 0.001.

To further evaluate the antimalarial activity of LZ1, we carried out in vivo anti-plasmodial assay in a *Plasmodium berghei* (*P. berghei*)-infected mouse model. Two murine animal models were performed (Figure 2A,B). In the classical four day suppression test (Figure 2C), the parasitemia rate was 49% in the vehicle mice on day four and was 39%, 35%, and 24% in 4, 8 and 12 mg·kg^−1^ LZ1-treated mice, respectively, while chloroquine treatment abolished the infection. In the Rane’s test (Figure 2D), the parasitemia rate increased in a time-dependent manner in both vehicle and LZ1-treated mice, while chloroquine-treatment abolished the infection after two days of administration. Even so, LZ1 slowed parasitemia growth rate and prolonged mice survival in a dose-dependent manner compared to the vehicle group (Figure 2E).

### 2.3. LZ1 Regulates Cytokines Expression and Liver Function

**Figure 3:**
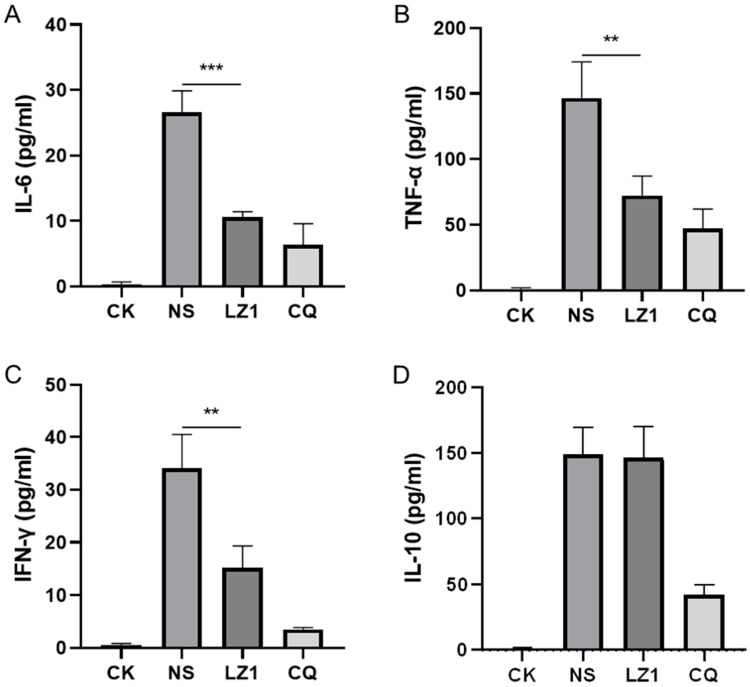
Effects of LZ1 and CQ on serum cytokines concentration of infected mice in four day suppression test. The concentrations of serum interleukin (IL)-6 (**A**), tumor necrosis factor (TNF)-α (**B**), interferon (IFN)-γ (**C**), and IL-10 (**D**) of mice were measured. CK and NS represent uninfected untreated and infected untreated mice, respectively. LZ1 represents infected mice treated with LZ1 at 12 mg·kg^−1^ body weight. CQ presents infected mice treated with chloroquine at 25 mg·kg^−1^ body weight. Data are presented as mean ± SEM (*n* = 6 mice per group). ** *p* < 0.01, *** *p* < 0.001.

Host-derived immunoregulation plays a crucial role in the pathogenesis of malaria in both humans and mice [17]. To further investigate the therapeutic implication of LZ1 in *P. berghei*-infected mice, we assessed several pro-inflammatory cytokines (interleukin (IL)-6, tumor necrosis factor (TNF)-α, interferon (IFN)-γ), and anti-inflammatory cytokine (IL-10) in serum specimens harvested in a four day suppression test. As illustrated in Figure 3, the levels of both pro-inflammatory cytokines and anti-inflammatory cytokine were elevated in the serum of *P. berghei*-infected mice compared to uninfected mice. However, LZ1 treatment significantly decreased the serum concentration of IL-6, TNF-α, and IFN-γcompared to the vehicle, while serum IL-10 concentration was still maintained at a high level.

**Figure 4:**
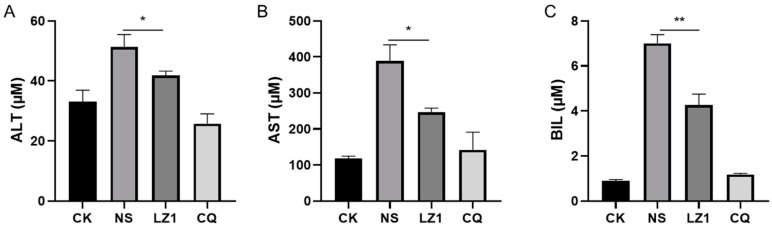
The liver function test of infected mice with LZ1 treatment in the four day suppression test. The concentrations of serum alanine transaminase (ALT) (**A**), aspartate transaminase (AST) (**B**), and bilirubin (BIL) (**C**) of mice were measured. CK and NS represent uninfected untreated and infected untreated mice, respectively. LZ1 represents infected mice treated with LZ1 at 12 mg·kg^−1^ body weight. CQ represents infected mice treated with chloroquine at 25 mg·kg^−1^ body weight. Data are presented as mean ± SEM (*n* = 6 mice per group). * *p* < 0.05, ** *p* < 0.01.

Liver injury is recognized as a clinical feature of malaria [18,19]. To determine whether LZ1 treatment affects liver function during malaria infection, we performed a liver function test by measuring the serum concentration of liver function biomarkers [20], including alanine transaminase (ALT), aspartate transaminase (AST), and total bilirubin (BIL). As illustrated in Figure 4, the serum contents of ALT, AST, and BIL were strongly elevated in *P. berghei*-infected mice. Compared with vehicle mice, a decrease of these biomarkers was observed in LZ1-treated mice, suggesting that LZ1 treatment alleviates liver function impairment and damage during malaria infection.

### 2.4. LZ1 Inhibits the Pyruvate Kinase Activity and ATP Synthesis During Plasmodium Infection

**Figure 5:**
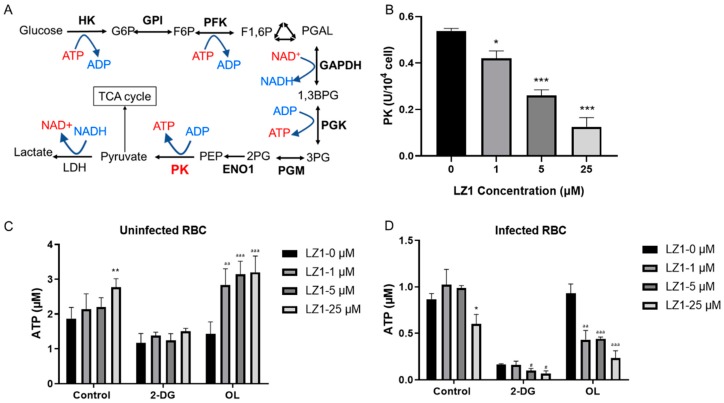
LZ1 inhibits the pyruvate kinase activity and adenosine triphosphate (ATP) synthesis during plasmodium infection. (**A**) Schematic of glycolysis pathway. Metabolic enzymes are bolded. PK, pyruvate kinase. (**B**) LZ1 inhibits the pyruvate kinase activity of infected erythrocyte. (**C**) 2-deoxyglucose (2-DG) induces ATP reduction in uninfected erythrocyte. (**D**) 2-DG induces ATP reduction in infected erythrocyte. Data are presented as mean ± SEM. * *p* < 0.05, ** *p* < 0.01, *** *p* < 0.001, ^#^ *p*< 0.05, ^aa^ *p* < 0.01, ^aaa^ *p* < 0.001.

Pyruvate kinase is critical for energy production in mature erythrocytes (Figure 5A), where mitochondria are absent and glycolysis is absolutely dominant in maintaining cell integrity and function [21]. In addition, glycolysis is the main pathway for adenosine triphosphate (ATP) production in malaria [22], and its deficiency shows a protective effect against malaria parasite [23,24]. To determine whether LZ1 affects the pyruvate kinase activity, we measured the enzymatic activity in RBCs from *P. berghei*-infected mice and uninfected mice (Figure 5B). The treatment of infected RBCs with LZ1 significantly suppressed the pyruvate kinase activity in a dose-dependent manner, whereas the pyruvate kinase activity was not affected in uninfected RBCs (data not shown). Collectively, these data indicated that LZ1 might inhibit the pyruvate kinase activity in malarial parasite-infected erythrocytes. 

Pyruvate kinase is a rate-limiting enzyme in glycolysis, catalyzing to generate one molecule of ATP by converting phosphoenolpyruvate to pyruvate. To identify whether LZ1 regulates cellular bioenergetics, we performed ATP assay by measuring cellular ATP content in RBCs infected with parasite or not. The RBCs were treated with 2-deoxyglucose (2-DG), a glycolysis inhibitor, and oligomycin (OL), which is an ATPase inhibitor that specifically inhibits mitochondrial oxidative phosphorylation [25]. ATP contents in uninfected RBCs were increased with the treatment of LZ1 and dropped to 58% of the control condition in the presence of 2-DG, while the dose-dependent increase in ATP levels was enhanced in the presence of OL (Figure 5C). However, treatment of infected RBCs with LZ1 showed a reduction in ATP content. With the treatment of LZ1, 2-DG reduced ATP content to 12% of control condition and OL reduced that to 42% (Figure 5D), suggesting that LZ1-induced reduction of ATP in infected RBCs was dependent on the glycolysis pathway. These data showed that ATP level increased when uninfected RBCs were treated with LZ1, whereas it dropped in infected RBCs, suggesting that LZ1 may induce decreased ATP of malaria parasite.

## 3. Discussion

Animal venoms are an invaluable natural resource of pharmacological tools that possess a large number of peptide toxins. Hundreds of peptide toxins from snake [26], scorpions [27], spiders [28], insects [29], and marine organisms [30] have been isolated or cloned, most of which exhibit anti-bacterial, anti-fungi, and anti-viral activities. Recently, a range of AMP toxins are emerging as new antimalarials, which show activities against malarial parasites in blood or mosquito stages. Although a variety of these peptides have antimalarial activity on cultured erythrocytic parasites, few have been shown to have in vivo antimalarial activity and no side effects on the host. Here, we present an AMP LZI derived from cathelicidin-BF that possesses antimalarial activity both in vitro and in vivo. 

The cationic (such as arginine and lysine) and the bulky (such as phenylalanine, valine, isoleucine, tryptophan, and tryptophan) amino acids are frequently included in these antimicrobial and antimalarial peptides [31]. LZ1 is composed of 15 amino acid residues with a linear structure and contains only four amino acids, including lysine, arginine, valine, and tryptophan. LZ1 showed both antimicrobial and antimalarial activities. During 72 h in vitro growth inhibition assay, an average ~61% reduction in parasite number was observed in the low-micromolar range of LZ1 (Figure 1). In vivo antimalarial activity of LZ1 was also confirmed in a murine malaria model (Figure 2). Several studies have revealed that cationic AMPs generally exert cytotoxicity. However, LZ1 showed little hemolytic activity and cytotoxicity to mammalian cells and did not interfere with normal physiological functions of mice [15]. Collectively, these results suggest LZ1 is a potential novel antimalarial peptide.

Cytokines have been reported to be associated with the pathophysiological processes of malaria. Some T helper 1 (Th1) cytokines (e.g., IFN-γ, lymphotoxin, and TNF) have been implicated in driving the immunopathological process leading to cerebral malaria, whereas some Th2 (e.g., IL-10, transforming growth factor (TGF)-β) cytokines appear to inhibit this process [32]. Our data showed that serum IL-6, TNF-α, IFN-γ, and IL-10 are elevated during *P. berghei* infection, and LZ1 exerted strong anti-inflammatory activity by inhibiting the overproduction of proinflammatory cytokines (IL-6, TNF-α, IFN-γ). A previous study has shown a strong positive association between the accumulation of inflammatory cytokines and the abnormality of liver function [33]. Despite the fact that liver injury was induced by *P. berghei* infection, LZ1 treatment decreased the serum concentration of liver function biomarkers (ALT, AST, and BIL), indicating that LZ1 potentially attenuates liver damage by suppressing the release of pro-inflammatory cytokines. 

A previous study indicated that some AMPs selectively disrupt the *P. gallinaceum* membrane and show harmless to erythrocytes [34]. A hypothetical model proposes that nonhemolytic peptides could undergo an affinity-drove transfer from the erythrocyte to the parasite membranes [35]. Based on this hypothesis, it is easy to explain that LZ1 selectively suppresses the pyruvate kinase activity in infected RBCs but not in normal RBCs. Pyruvate kinase activity is higher in infected RBCs than normal erythrocytes [36], as shown in Figure 5, and suppression of glycolysis by 2-DG in infected RBCs is much stronger than that in uninfected RBCs. ATP production in glycolysis is critical to the lifespan of RBCs [37], but the mechanism of LZ1 on ATP production in glycolysis of infected erythrocyte is still unclear, thus raising a need for further experiments. Although pyruvate kinase deficiency plays a protective role against malaria, this deficiency leading to ATP depletion in RBCs affects the viability of erythrocyte [21] and is considered the most common defect of the glycolytic pathway that leads to congenital hemolytic anemia [38]. LZ1 here acted selectively on infected RBCs to reduce cellular ATP content, which supports its potential as a candidate for treating malaria parasite by inhibiting pyruvate kinase.

## 4. Conclusions

In conclusion, the present study demonstrates the in vitro and the in vivo antimalarial activity of LZ1, a peptide derived from cathelicidin-BF. LZ1 might have a dual mechanism for protection against malaria parasite: (1) exerting anti-inflammatory effects during *P. berghei* infection; (2) inhibiting the ATP production in the glycolytic pathway of parasite-infected erythrocyte. These findings highlight that LZ1 has the potential for the development of novel antimalarials to complement current therapeutic strategies aimed at combating drug resistance among malaria parasites.

## 5. Materials and Methods

### 5.1. Animals and Parasite

Male Kunming mice were purchased from the Animal Center, Kunming Medical College (Yunnan, China). The mice (8 weeks old) were housed in standard mouse cages at 20–25 °C, 70% relative humidity, and under a 12 h-light and 12 h-dark cycle with free access to food and water. All animal experiments were approved by the Institutional Review Board and the Animal Care and Use Committee at Kunming Institute of Zoology (identification code: SMKX-2018028; date of approval: 26 May 2017). 

*P. falciparum* line 3D7 was acquired from Malaria Research and Reference Reagent Resource Center (Manassas, VA, USA). In vitro culture of *P. falciparum* was carried out following standard methods with modifications as described [39].

*P. berghei* ANKA parasites were obtained from Xinxiang Medical University (Xinxiang, Henan, China). The parasites were usually maintained frozen at −80 °C. An aliquot of frozen parasites was thawed and injected intraperitoneally into donor mice. Donor mice infected with 30% parasitemia were sacrificed, and blood was used to infect mice in the antimalarial test.

### 5.2. Peptides Synthesis

LZ1 (VKRWKKWWRKWKKWV-NH_2_) was synthesized by GL Biochem Ltd. (Shanghai, China) and analyzed by reversed phase high performance liquid chromatography (RP-HPLC) and mass spectrometry to confirm the purity to be higher than 98%.

### 5.3. In Vitro Antimalarial Assay

A 72 h in vitro growth inhibition assay [40,41] was used to test the antimalarial activity of LZ1. *P. falciparum* line 3D7 was cultured in complete RPMI-1640 medium with 2% hematocrit and incubated in a humidified atmosphere with 5% O_2_, 5% CO_2_, and 90% N_2_ at 37 °C. The parasites were synchronized by treating with 5% sorbitol (Sigma, Burlington, MA, USA) when most of them were in the ring stage. Two hundred microliters of the culture with a parasitaemia of 0.5% ring parasites and a hematocrit of 2% was added into a 96-well plate with a known LZ1 concentration (1 μM, 5 μM, and 25 μM). The antimalarial effect of the LZ1 was estimated 48 h after treatment with it, and the parasite re-invaded red blood cells at the ring stage. The live parasites of thin blood films stained with Giemsa (Sigma, Burlington, MA, USA) were counted under a microscope. Total parasitaemia of treated cultures was compared to the parasites cultured in the absence of the peptide. Experiments were carried out by triplicate.

### 5.4. In Vivo Antimalarial Assay

The in vivo antimalarial activity of LZ1 was assessed by the classical 4 day suppression test [42] according to our previous report [43]. Male Kunming mice were infected by intravenous (i.v.) inoculation of 1.0 × 10^6^ infected erythrocytes from donor mice on the first day (day 0) of the experiment. The mice were randomly allocated to five groups with six mice in each group. Peptide-treatment (4, 8, or 12 mg/kg body weight of LZ1) was administrated within 1 h post-inoculation of mice with the parasite (day 0) in a dose volume of 0.1 mL. Chloroquine diphosphate (Sigma, Burlington, MA, USA) (25 mg/kg body weight) and normal saline (equal volume) were used as positive and negative controls, respectively. Mice were dosed daily by i.v. injection for 4 consecutive days. On day 4, serum was collected, and tail blood smear was taken, stained with 10% Giemsa for 15 min, and examined under a microscope at 100×. The percentage of parasitemia was determined by counting parasitized RBCs on at least 3000 cells. Rane’s test [44] was performed for the assessment of survivals. Five groups of infected mice were dosed daily on day 3 to day 6 by i.v. injection. The percentage of parasitaemia was calculated daily on day 4 to day 7. Survivals were followed up until day 21 post-infection.

### 5.5. Measurement of Serum Cytokines and Liver-Function

Mice serum was collected in the 4 day suppression test. The concentrations of serum cytokines (IL-10, IL-6, IFN-γ, and TNF-α) were determined by enzyme-linked immunosorbent assays (ELISA) (Dakewe, Shenzhen, Guangdong, China). The liver-function test was performed by detecting the serum content of ALT, AST, and BIL (Roche Diagnostics GmbH, Mannheim, Germany) by using automatic biochemistry analyzer (COBAS INTEGRA^®^ 400 plus, Roche, Mannheim, Germany) according to the manufacturer’s instructions.

### 5.6. ATP Assays

Intracellular ATP concentration was measured with ATP Assay Kit (Beyotime, Shanghai, China) according to the manufacturer’s directions. Briefly, RBCs were seeded in a 48-well plate at 2.5 × 10^5^ cells per well and incubated with LZ1, 2-DG (Aladdin, Shanghai, China), or OL (CST, Beverly, MA, USA). After 4 h of drug exposure, cells were lysed, and supernatants were mixed with assay reagent. The mixtures were transferred into opaque-walled 96-well plates (3603, Costar), and luminescence was read by microplate reader (Flexstation^®^ 3, Molecular Devices, San Jose, CA, USA). The ATP concentration of treated RBCs was determined by comparison to the luminescence values of an ATP standard curve.

### 5.7. Pyruvate Kinase Activity Assay

Pyruvate kinase activity was measured by Pyruvate Kinase Assay Kit according to the manufacturer’s instructions. Briefly, RBCs from mice infected with *P. berghei* were seeded in a 24-well plate at 2.5 × 10^6^ cells per well and incubated with serially diluted LZ1 (1, 5, and 25 μM) for 4 h. RBCs were lysed by sonication, and cell lysates were centrifuged at 16,000 g at 4 °C for 10 min. Phosphoenolpyruvate and ADP were catalyzed by pyruvate kinase in the supernatant to generate pyruvate and ATP. The produced pyruvate was coupled to oxidation of nicotinamide adenine dinucleotide (NADH) by lactic dehydrogenase (LDH). The decrease in NADH was measured at OD 340 nm for 5 min with a spectrophotometer (Flexstation 3, Molecular Devices, San Jose, CA, USA), and the slopes of the curves were used as a measure of pyruvate kinase activity.

### 5.8. Statistical Analysis

Statistical analysis was conducted with GraphPad Primer 8.0 (GraphPad Software Inc., GraphPad Prism 8.0.1.244, San Diego, CA, USA, 2018). Data were analyzed statistically using one-way ANOVA and two-tailed Student’s t-test to identify the differences between the treated group and the controls. All data are presented as means ± SEM. A value of *p* < 0.05 was considered significant.
